# *Staphylococcus aureus* single-stranded DNA-binding protein SsbA can bind but cannot stimulate PriA helicase

**DOI:** 10.1371/journal.pone.0182060

**Published:** 2017-07-27

**Authors:** Yen-Hua Huang, Hong-Hsiang Guan, Chun-Jung Chen, Cheng-Yang Huang

**Affiliations:** 1 School of Biomedical Sciences, Chung Shan Medical University, Taichung City, Taiwan; 2 Life Science Group, Scientific Research Division, National Synchrotron Radiation Research Center, Hsinchu, Taiwan; 3 Institute of Biotechnology, and University Center for Bioscience and Biotechnology, National Cheng Kung University, Tainan City, Taiwan; 4 Department of Physics, National Tsing Hua University, Hsinchu, Taiwan; 5 Department of Medical Research, Chung Shan Medical University Hospital, Taichung City, Taiwan; Istituto di Genetica Molecolare, ITALY

## Abstract

Single-stranded DNA-binding protein (SSB) and PriA helicase play important roles in bacterial DNA replication restart process. The mechanism by which PriA helicase is bound and stimulated by SSB in *Escherichia coli* (Ec) has been established, but information on this process in Gram-positive bacteria are limited. We characterized the properties of SSB from *Staphylococcus aureus* (SaSsbA, a counterpart of EcSSB) and analyzed its interaction with SaPriA. The gel filtration chromatography analysis of purified SaSsbA showed a stable tetramer in solution. The crystal structure of SaSsbA determined at 1.82 Å resolution (PDB entry 5XGT) reveals that the classic oligonucleotide/oligosaccharide-binding folds are formed in the N-terminal DNA-binding domain, but the entire C-terminal domain is disordered. Unlike EcSSB, which can stimulate EcPriA via a physical interaction between EcPriA and the C-terminus of EcSSB (SSB-Ct), SaSsbA does not affect the activity of SaPriA. We also found that SaPriA can be bound by SaSsbA, but not by SaSsbA-Ct. Although no effect was found with SaSsbA, SaPriA can be significantly stimulated by the Gram-negative *Klebsiella pneumoniae* SSB (KpSSB). In addition, we found that the conserved SSB-Ct binding site of KpPriA (Trp82, Tyr86, Lys370, Arg697, and Gln701) is not present in SaPriA. Arg697 in KpPriA is known to play a critical role in altering the SSB_35_/SSB_65_ distribution, but this corresponding residue in SaPriA is Glu767 instead, which has an opposite charge to Arg. SaPriA E767R mutant was constructed and analyzed; however, it still cannot be stimulated by SaSsbA. Finally, we found that the conserved MDFDDDIPF motif in the Gram-negative bacterial SSB is DISDDDLPF in SaSsbA, i.e., F172 in EcSSB and F168 in KpSSB is S161 in SaSsbA, not F. When acting with SaSsbA S161F mutant, the activity of SaPriA was dramatically enhanced elevenfold. Overall, the conserved binding sites, both in EcPriA and EcSSB, are not present in SaPriA and SaSsbA, thereby no stimulation occurs. Our observations through structure-sequence comparison and mutational analyses indicate that the case of EcPriA-EcSSB is not applicable to SaPriA-SaSsbA because of inherent differences among the species.

## Introduction

Single-stranded DNA (ssDNA)-binding protein (SSB) is essential for DNA metabolic processes [1, 2]. The binding of SSB to ssDNA is independent of the sequence of DNA. SSB protects susceptible ssDNA from nucleolytic digestion and prevents the secondary structure formation of ssDNA [3]. In addition, SSB also binds to many DNA-binding proteins that constitute the SSB interactome [2, 4, 5]. The functions of SSB are well studied in *Escherichia coli* (EcSSB), and relatively little is known about SSB from other bacteria. SSBs are typically homotetramers [3, 6, 7], in which four oligonucleotide/oligosaccharide-binding folds (OB folds) form a DNA-binding domain [8–12]. However, SSB from the bacterial phylum *Deinococcus*-*Thermus* functions as a homodimer, in which each monomer contains two OB folds linked by a conserved spacer sequence [13–20]. SSB from *Sulfolobus solfataricus* is a monomer (one OB fold) [21–25]. Recently, a distinct SSB ThermoDBP is found to bind ssDNA without the classical OB folds of bacterial SSB [26, 27].

Bacterial SSBs consist of a conserved N-terminal ssDNA-binding/oligomerization domain (SSBn) and a flexible, highly disordered C-terminal protein–protein interaction domain (SSBc) [2, 28]. SSBc can be further subdivided into two sub-domains: a long proline- or glycine-rich hinge, also known as the intrinsically disordered linker (IDL) and, the highly conserved acidic tail of the last six C-terminal amino acid residues of SSB (DDDIPF) [2, 29]. This acidic tail of SSB binds to many DNA-binding proteins, and the activities of some of these proteins are stimulated by their interactions with ssDNA-bound SSB [2]. The binding of SSB to ssDNA makes IDL easily accessible to other proteins, such as proteases and DNA polymerase III [30, 31]. The C-terminus in SSB can also interact with the OB fold and regulate the ssDNA-binding activity of SSB itself [32, 33]. EcSSB has two major ssDNA binding modes [34]. In the (SSB)_35_-binding mode, two subunits of the tetramer participate in ssDNA binding, whereas in the (SSB)_65_-binding mode, all four subunits interact with ssDNA. The binding mode is dependent on the concentrations of protein and salt in the solution. During the different stages of DNA metabolism, different binding modes of SSB to ssDNA may be required for the in vivo function [35–37].

PriA is a DEXH-type helicase, utilized during replication restart to reload DnaB back onto the chromosome [38, 39]. Fuelled by the binding and hydrolysis of ATP, PriA moves along the nucleic acid filaments with other primosomal proteins and separates double-stranded DNA into their complementary single strands [40–42]. In *E*. *coli*, the replication restart primosome consists of PriA, PriB, PriC, DnaB helicase, DnaC, DnaT, and DnaG primase [38, 39, 43]. PriA recognizes stalled DNA replication forks with either duplex or SSB-coated ssDNA lagging strands and then processes for full primosome assembly [43–45]. However, PriA is a poor helicase when acting alone and might need other accessory proteins, such as PriB and SSB, to stimulate the helicase activity [46, 47]. The reaction mechanisms of DNA replication restart primosome are well studied in Gram-negative *E*. *coli* [43, 44, 48–53]. Relatively little is known about the regulation of the Gram-positive bacterial PriA-directed primosome activity [54, 55]. In the Gram-positive *Bacillus subtilis*, the initiator protein PriA helicase has a homolog of *E*. *coli* [56]. Nevertheless, PriB, PriC, DnaT, and DnaC proteins are not found in Gram-positive bacteria. Instead, Gram-positive *B*. *subtilis* has different primosomal proteins, namely, DnaD, DnaB, and DnaI, which are essential for replication restart [57]. Whether SSB functions and participates in the Gram-positive bacterial PriA-directed primosome assembly in a manner different from that of *E*. *coli* is still unknown.

In this study, we have cloned, expressed, purified, and crystallized the Gram-positive *Staphylococcus aureus* main SSB (SaSsbA) and determined its structure at 1.82 Å resolution. Basing on the results from surface plasmon resonance (SPR) experiments, ATPase stimulation effects and structure-sequence analysis on SaSsbA–SaPriA interaction, we found that SaSsbA can bind but cannot stimulate SaPriA. Through domain deletion and structure-based mutational analyses, we conclude that the conserved binding sites, both in EcPriA and EcSSB, are not present in SaPriA and SaSsbA, thereby no stimulation occurs.

## Materials and methods

### Construction of plasmids for SaSsbA, tag-free SaSsbA, KpSSB, tag-free KpSSB, KpSSBc, SaDnaD, and SaPriA expression

Construction of the SaDnaD [58], SaPriA [59], *Klebsiella pneumoniae* SSB (KpSSB) [60], and tag-free KpSSB [61] expression plasmids has been reported. The gene encoding SaSsbA (the accession number ACY10277) was amplified by PCR using the genomic DNA of *S*. *aureus* subsp. *aureus* ED98 as template. The forward and reverse primers were designed to introduce unique restriction sites into SaSsbA, permitting the insertion of the amplified gene into the pET21b vector (Novagen Inc., Madison, WI, USA) for protein expression in *E*. *coli*. To obtain His tag-free SaSsbA, a fragment containing the coding sequence of SaSsbA and the stop codon was directly amplified and ligated into the pET21b vector. KpSSBc (aa 116–174) was also subcloned in the pET21b vector. Primers used for construction of these plasmids are summarized in Table 1.

**Table 1:** Primers used for construction of plasmids.

Oligonucleotide	Primer
SaSsbA-NdeI-N	GGGCATATGCTAAATAGAGTTGTATTA
SaSsbA-XhoI-C	CCATTCTCGAGGAATGGTAAGTCATCA
Tag-free SaSsbA-NdeI-N	GGGCATATGCTAAATAGAGTTGTATTA
Tag-free SaSsbA-XhoI-C	CCATTCTCGAGTTAGAATGGTAAGTCATCA
KpSSBc-NdeI-N	GGGCATATGCGTCAGGGCGGCGGCGCACCG
KpSSBc-XhoI-C	GGGCTCGAGGAACGGGATGTCGTCGTCGAA
SaPriA E767R-N	TATAAAAGTGAACGTGGATTATTACAAGCC
SaPriA E767R-C	TTGTAATAATCCACGTTCACTTTTATATTT
SaPriA R434A-N	GAAAGTTATGCAGCAGCTGAAAAAGACGTT
SaPriA R434A-C	GTCTTTTTCAGCTGCTGCATAACTTTCAAG
SaSsbA S161F-N	GGACCGATTGATATATTCGATGATGACTTACCA
SaSsbA S161F-C	GTCATCATCGAATATATCAATCGGTCCGTTTGC
SaSsbA S161F/delI160-N	GGACCGATTGATTTCGATGATGACTTACCA
SaSsbA S161F/delI160-C	TAAGTCATCATCGAAATCAATCGGTCCGTT

These plasmids were verified by DNA sequencing. Underlined nucleotides indicate the designated site for the restriction site or the mutation site.

### Protein concentration

The protein concentration of the solutions was determined by the Bio-Rad Protein Assay using bovine serum albumin as a standard (Bio-Rad, CA, USA). The Bio-Rad Protein Assay is a dye-binding assay in which a differential color change of a dye occurs in response to various concentrations of protein [62].

### Protein expression and purification

Purification of the recombinant SaDnaD [58], SaPriA [59], KpSSB [60], and tag-free KpSSB [61] has been reported. The recombinant SaSsbA and KpSSBc were expressed and purified using the protocol described previously for PriB [63]. Briefly, *E*. *coli* BL21(DE3) cells were transformed with the expression vector and overexpression of the expression plasmids was induced by incubating with 1 mM isopropyl thiogalactopyranoside. The protein was purified from the soluble supernatant by Ni^2+^-affinity chromatography (HiTrap HP; GE Healthcare Bio-Sciences), eluted with Buffer A (20 mM Tris-HCl, 250 mM imidazole, and 0.5 M NaCl, pH 7.9), and dialyzed against a dialysis buffer (20 mM HEPES and 100 mM NaCl, pH 7.0; Buffer B). Protein purity remained at >97% as determined by SDS-PAGE (Mini-PROTEAN Tetra System; Bio-Rad, CA, USA).

The recombinant tag-free SaSsbA was expressed and purified using the protocol described previously [61] for *Pseudomonas aeruginosa* SSB (PaSSB) and *Salmonella enterica* serovar Typhimurium LT2 SSB (StSSB) with the following modifications. The cells overexpressing the protein were chilled on ice, harvested by centrifugation, resuspended in Buffer C (20 mM Tris-HCl and 50 mM NaCl, pH 7.9) and disrupted by sonication with ice cooling. The protein solution (50 mL) was precipitated from the supernatant of the cell lysate by incubation with 0.27 g/mL of ammonium sulfate for 30 min and centrifugation at 20000g for 10 min. The pellets were washed twice with 2.0 mL of Buffer D (20 mM Tris-HCl, 50 mM NaCl, and 1.2 M ammonium sulfate, pH 7.9). After dialysis against Buffer C, the protein solution applied to the Q column (GE Healthcare Bio-Sciences, Piscataway, NJ, USA) was eluted with a linear NaCl gradient from 0.1 to 0.6 M with Buffer C using the AKTA-FPLC system (GE Healthcare Bio-Sciences, Piscataway, NJ, USA). The peak fractions with the ssDNA binding activity were collected and dialyzed against Buffer E (20 mM potassium phosphate, 1 mM EDTA, and 100 mM NaCl, pH 7.0). The protein solution was then applied to the Heparin HP column (GE Healthcare Bio-Sciences, Piscataway, NJ, USA) and eluted with a linear NaCl gradient from 0.1 to 1.0 M with Buffer E. The peak fractions from this chromatographic step with the ssDNA binding activity were collected and concentrated, and the purity of tag-free SaSsbA remained at >97% as determined by SDS-PAGE.

### Gel-filtration chromatography

Gel-filtration chromatography was carried out by the AKTA-FPLC system (GE Healthcare Bio-Sciences, Piscataway, NJ, USA). In brief, purified SaSsbA (2 mg/mL) in Buffer B was applied to a Superdex 200 prep grade column (GE Healthcare Bio-Sciences, Piscataway, NJ, USA) equilibrated with the same buffer. The column was operated at a flow rate of 0.5 ml/min, and 0.5-ml fractions were collected. The proteins were detected by measuring the absorbance at 280 nm. The column was calibrated with proteins of known molecular weight: thyroglobulin (670 kDa), γ-globulin (158 kDa), albumin (67 kDa), ovalbumin (43 kDa), chymotrypsinogen A (25 kDa) and ribonuclease A (13.7 kDa). The K_av_ values for the standard proteins and SaSsbA were calculated from the equation: K_av_ = (V_e_−V_o_)/(V_c_−V_o_), where V_o_ is column void volume, V_e_ is elution volume, and V_c_ is geometric column volume.

### Electrophoretic mobility shift assay (EMSA)

EMSA for SaSsbA was conducted using the protocol described previously for SSB [64]. Briefly, radiolabeling of various lengths of ssDNA oligonucleotides was carried out with [γ^32^P]ATP (6000 Ci/mmol; PerkinElmer Life Sciences, Waltham, MA) and T4 polynucleotide kinase (Promega, Madison, WI, USA). The protein (0, 0.037, 0.075, 0.15, 0.31, 0.62, 1.25, 2.5, 5, and 10 μM; monomer) was incubated for 30 min at 25°C with 1.7 nM DNA substrates in a total volume of 10 μL in 20 mM Tris-HCl pH 8.0 and 100 mM NaCl. Aliquots (5 μl) were removed from each of the reaction solutions, and added to 2 μl of gel-loading solution (0.25% bromophenol blue and 40% sucrose). The resulting samples were resolved on a native 8% polyacrylamide gel at 4°C in TBE buffer (89 mM Tris borate and 1 mM EDTA) for 1 h at 100 V, and were visualized by phosphorimaging. The phosphor storage plate was scanned, and the data for complex and free DNA bands were digitized for quantitative analysis. The ssDNA binding ability for the protein was estimated using linear interpolation from the protein concentration that binds 50% of the input DNA. Each [Protein]_50_ was calculated as the average of at least three measurements ± S.D.

### Preparation of dsDNA substrate

The double-stranded DNA substrates (dsDNA) were prepared with a radiolabeled PS4 strand (3'-GGGCTTAAGCCTATCGAGCCATGGG-5'; 25 mer) and an unlabeled PS3-dT30 strand (5'-CCCGAATTCGGATAGCTCGGTACCC-dT30-3') at a 1:1 concentration ratio. Each dsDNA substrate was formed in 20 mM HEPES (pH 7.0) and 100 mM NaCl, by brief heating at 95°C for 5 min and then followed by slow cooling to room temperature overnight.

### Surface plasmon resonance (SPR)

SPR was conducted using the protocol described previously for DnaC helicase [65]. SaPriA was immobilized on Series S sensor chips CM5 (GE Healthcare Bio-Sciences, Piscataway, NJ, USA). The SaPriA-binding experiments were carried out at 293K using a Biacore T200 (GE Healthcare Bio-Sciences, Piscataway, NJ, USA) with running buffer (40 mM Tris, 200 mM NaCl, and 0.05% Tween-20 at pH 8.0). SaSsbA solutions were diluted in the running buffer to final concentrations of 1000, 500, 250, 125, and 63 nM. The Diluted samples were injected in duplicate over the immobilized protein for 120 s at a flow rate of 30 μL/min. The running buffer was then flushed for 300 s at a flow rate of 30 μl/min. Finally, the chip surface was regenerated by injecting 2 M MgCl_2_ buffer for 60 s at a flow rate of 30 μl/min. Control samples were used to monitor the sensor chip surface stability, demonstrating reproducibility throughout the duration of the experiments. The estimated *K*_d_ values were derived by fitting the association and dissociation signals with a 1:1 (Langmuir) model using the Biacore T200 Evaluation Software. Chemically synthesized peptides SaSsbA-Ct (NANGPIDISDDDLPF) and KpSSB-Ct (PSNEPPMDFDDDIPF) were also used (4–7 different concentrations ranging from 0.06 to 2 μM) for SaPriA-binding experiments.

### ATPase assay

SaPriA ATPase assay [58] was performed with 0.4 mM [γ-^32^P] ATP and 0.12 μM of SaPriA in reaction buffer containing 40 mM Tris (pH 8.0), 10 mM NaCl, 2 mM DTT, 2.5 mM MgCl_2_, and 0.1 μM PS4/PS3-dT30 DNA substrate. Aliquots (5 μL) were taken and spotted onto a polyethyleneimine cellulose thin-layer chromatography plate, which was subsequently developed in 0.5 M formic acid and 0.25 M LiCl for 30 m. Reaction products were visualized by autoradiography and quantified with a phosphorimager.

### Crystallography

Before crystallization, SaSsbA was concentrated to 20 mg/mL in Buffer B. Crystals were grown at room temperature by hanging drop vapor diffusion in 22% PEG 4000, 100 mM HEPES, 100 mM sodium acetate, pH 7.5. Data were collected using an ADSC Quantum-315r CCD area detector at SPXF beamline BL13C1 at NSRRC (Taiwan, ROC). All data integration and scaling were carried out using HKL-2000 [66]. There were two SaSsbA monomers per asymmetric unit. The crystal structure of SaSsbA was solved at 1.82 Å resolution with the molecular replacement software AMoRe [67] using *Mycobacterium smegmatis* SSB as model (PDB entry 1X3E). After molecular replacement, model building was carried out using XtalView [68]. CNS was used for molecular dynamic refinement [69]. The final structure was refined to an *R*-factor of 0.1932 and an *R*_free_ of 0.2233. Atomic coordinates and related structure factors have been deposited in the PDB with accession code 5XGT.

### Bioinformatics

The amino acid sequences of 417 sequenced PriA and 484 SSB homologs were aligned using ConSurf [70]. The model of SaPriA was built from the coordinates of 4NL4 (crystal structure of KpPriA) by using SWISS-MODEL, http://swissmodel.expasy.org/. The structures were visualized by using the program PyMol.

## Results

### Analysis of the *ssb* (*SAAV_0334*) gene

**Fig 1:**
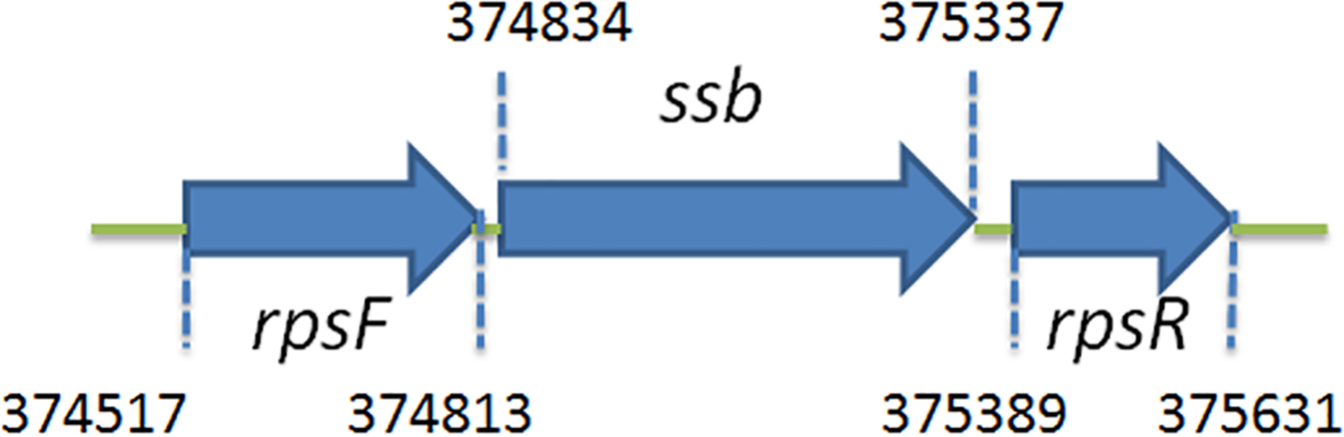
Gene map of *S*. *aureus* chromosomal region with *ssb*. The gene *SAAV_0334* coding for SSB (designated as SsbA in this study) maps from the 374834 to 375337 nt of the *S*. *aureus* genome. This *ssb* gene is flanked by the *rpsF* and *rpsR* genes, coding for the ribosomal proteins S6 and S18, respectively.

Based on the similar nucleotide sequence to *E*. *coli* SSB, the gene *SAAV_0334*, which encodes *S*. *aureus* main SSB, was initially found using a database search through the National Center for Biotechnology Information (NCBI). In this study, this SSB was designated as SsbA. Fig 1 shows the map of *S*. *aureus*, in which the *ssb* gene is flanked by the *rpsF* and *rpsR* genes, coding for the ribosomal proteins S6 and S18, respectively. Unlike *E*. *coli ssb* gene organization, *S*. *aureus* and *B*. *subtilis ssb* genes are not located adjacent to *uvrA* gene. These genes (*rpsF*, *ssb*, and *rpsR*) in *B*. *subtilis* belong to one operon and are controlled by the SOS response [71].

### Sequence analysis of SaSsbA

**Fig 2:**
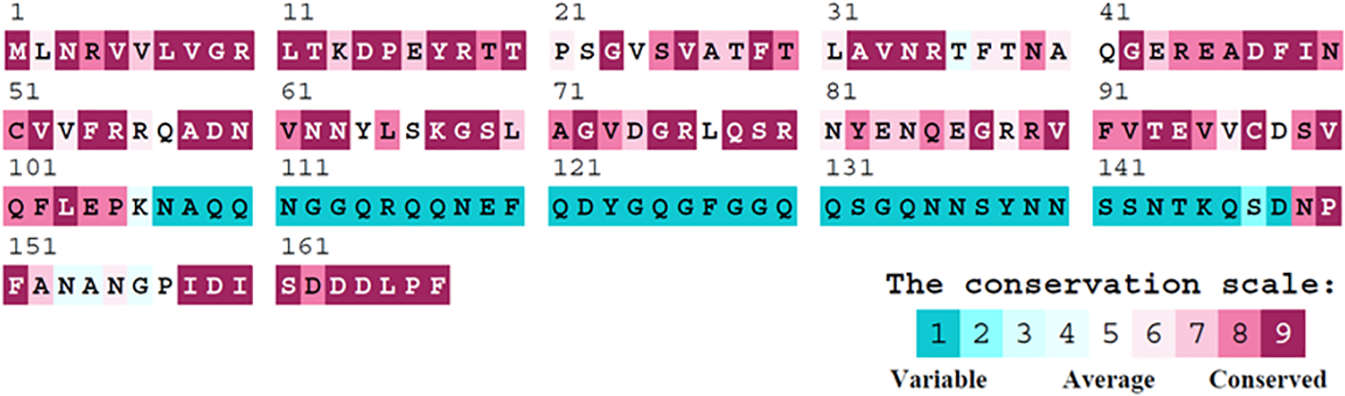
Sequence analysis of SaSsbA. An alignment consensus of 484 sequenced SSB homologs by ConSurf reveals the degree of variability at each position along the sequence. Highly variable amino acid residues are colored teal, whereas highly conserved amino acid residues are burgundy. A consensus sequence was established by determining the most commonly found amino acid residue at each position relative to the sequence of SaSsbA.

The gene *SAAV_0334*, which encodes *S*. *aureus* SsbA (SaSsbA), was initially found using a database search through NCBI. Based on the known nucleotide sequence, the predicted SaSsbA monomer protein has a length of 167 amino acid residues and a molecular mass of 19 kDa. Analysis of the sequence of SaSsbA by RPS-BLAST showed the presence of a putative OB-fold domain that is common in all known SSBs. Fig 2 shows the alignment consensus of 484 sequenced SSB homologs by ConSurf [70], revealing the degree of variability at each position along the sequence. In the EcSSB–ssDNA complex [72], four essential aromatic residues, Trp40, Trp54, Phe60, and Trp88, conserved in most SSB families as Phe/Tyr/Trp, participate in ssDNA binding via stacking interactions. The corresponding residues in SaSsbA are Phe37, Phe48, Phe54, and Tyr82; no Trp residue was observed in SaSsbA. The important C-terminal tail DDDIPF of EcSSB involved in protein–protein interaction is DDDLPF in SaSsbA.

### Sequence analysis of SaPriA

**Fig 3:**
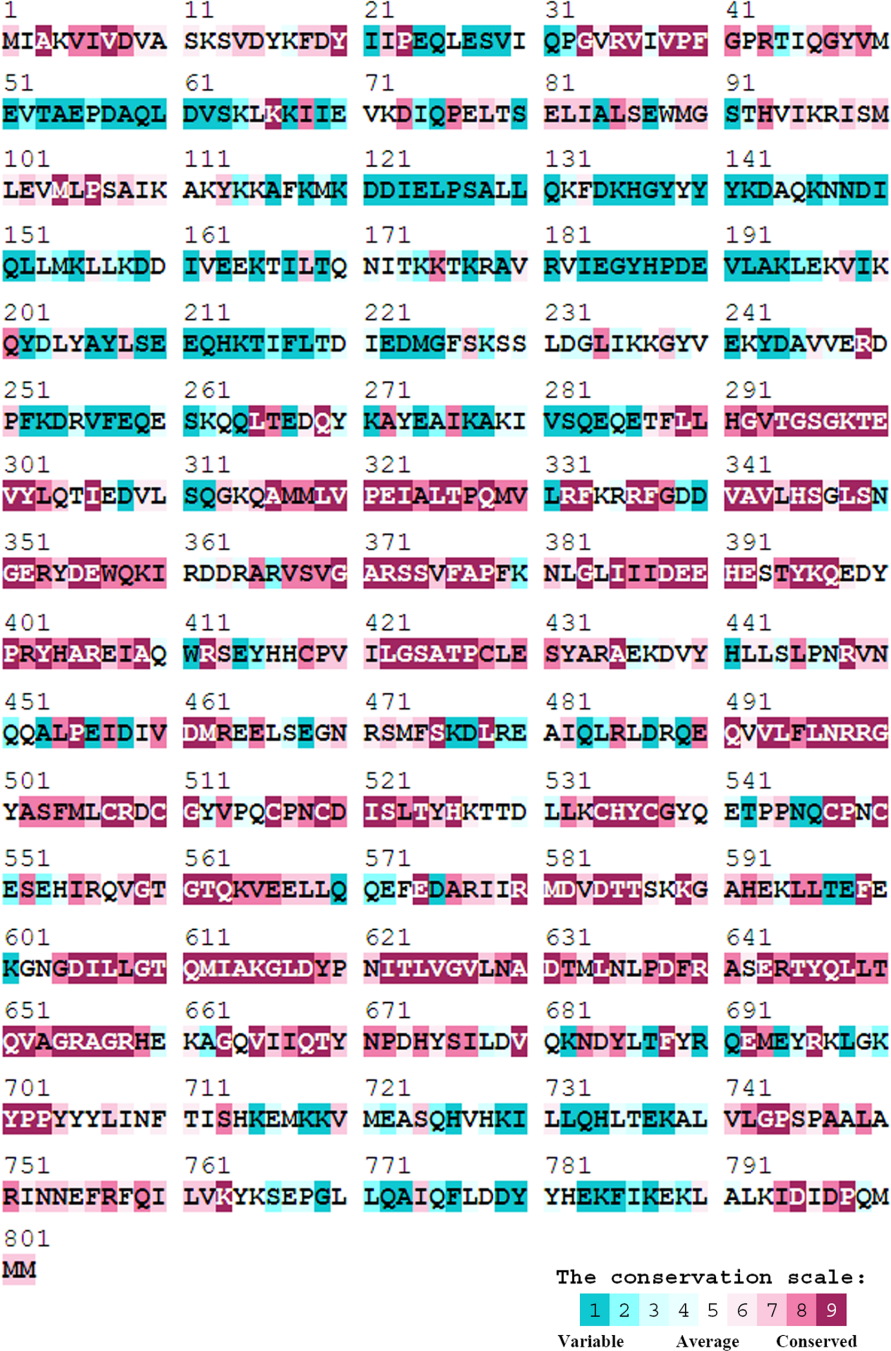
Sequence analysis of SaPriA. An alignment consensus of 417 sequenced PriA homologs by ConSurf reveals the degree of variability at each position along the sequence. In general, amino acid residues in the C-terminal region of PriA are conserved.

Based on the known nucleotide sequence, the predicted SaPriA monomer protein has a length of 802 amino acid residues (which is higher than that for EcPriA with 732 amino acid residues) and a molecular mass of 92.7 kDa, with a pI of 6.12. Analysis of the structure of SaPriA indicated a DEAD-like helicase (aa 288–427) and revealed the presence of the putative Mg^2+^ ion binding site (aa 388–391), ATP-binding sites (aa 295–299, 618, 651, 655, and 658), and nucleotide-binding region (580, 581, and 610–612). Fig 3 shows that the alignment consensus of 417 sequenced PriA homologs by ConSurf revealed the degree of variability at each position along the sequence, in which the binding sites mentioned above are highly conserved among varying organisms. Amino acid residues in the C-terminal helicase domain of PriA are also conserved. However, amino acid residues in the N-terminal region of SaPriA, especially aa 114–286, are variable. Given that distinct mechanisms exist for reloading the replicative DnaB helicase by the Gram-positive and Gram-negative bacterial PriA, whether or not this highly variable region (aa 114–286) in SaPriA is responsible for binding by the Gram-positive bacterial specific helicase loader is still unknown.

### Purification of SaSsbA and tag-free SaSsbA

The gene *SAAV_0334* encoding for the putative SaSsbA was PCR-amplified using the genomic DNA of *S*. *aureus* subsp. *aureus* ED98 as template. This amplified gene was then ligated into the pET21b vector for protein expression. SaSsbA with a Hig tag was heterologously overexpressed in *E*. *coli* and then purified from the soluble supernatant by Ni^2+^-affinity chromatography. Pure protein was obtained in this single chromatographic step with an elution of Buffer A and dialyzed against a dialysis buffer (Buffer B). Approximately >10 mg of purified protein was obtained from 1 L of *E*. *coli* cell culture. To exclude the possible effect of a His tag, tag-free SaSsbA was also produced and purified by precipitation of ammonia sulfate, Q, and Heparin column chromatographies. Approximately 2 mg of purified tag-free SaSsbA was obtained from 1 L of *E*. *coli* cell culture.

### SaSsbA bound to ssDNA

**Fig 4:**
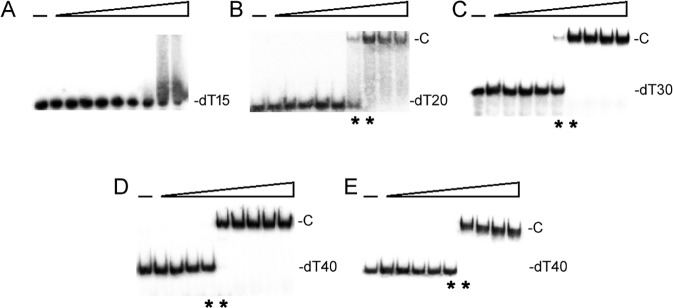
EMSA of SaSsbA. Protein (0, 0.037, 0.075, 0.15, 0.31, 0.62, 1.25, 2.5, 5, and 10 μM; monomer) was incubated at 25°C for 30 min with 1.7 nM of (A) dT15, (B) dT20, (C) dT30, or (D) dT40 in a total volume of 10 μL in 20 mM Tris—HCl (pH 8.0) and 100 mM NaCl. (E) The binding of SaSsbA to dT40 in the presence of 0.4 M NaCl. The [Protein]_50_ values of SaSsbA as a function of the length of the ssDNA were determined using EMSA. Protein concentrations used to determine the midpoint values are indicated by asterisks.

To investigate the length of nucleotides needed for the formation of the SaSsbA–ssDNA complex, as well as the ssDNA-binding ability of SaSsbA, we studied the binding of SaSsbA to dT15, dT20, dT30, and dT40 with different protein concentrations (Fig 4). The binding ability of SaSsbA to dT40 in the presence of 0.4 M NaCl was also analyzed (Fig 4). As shown in Fig 4A, no significant band shift was observed when SaSsbA was incubated with dT15, indicating that SaSsbA could not form a stable complex with this homopolymer. By contrast to dT15, longer dT homopolymers, which include dT20–40, produced a significant band shift (C, complex), i.e., formation of a stable protein–DNA complex in solution. To compare the binding abilities of SaSsbA with ssDNA of different lengths, the midpoint values for input ssDNA binding that were calculated from the titration curves of EMSA and the [Protein]_50_ values were quantified using linear interpolation from the protein concentration and are summarized in Table 2. The [SaSsbA]_50_ for dT40 binding is 90 ± 5 nM, which is twofold lower than that in the presence of 0.4 M NaCl (180 ± 22 nM).

**Table 2:** The [Protein]_50_ values of SaSsbA as analyzed by EMSA.

DNA	[Protein]_50_ (nM)
dT15	> 2000
dT20	292 ± 28
dT30	186 ± 20
dT40	90 ± 5
dT40 (with 0.4 M NaCl)	180 ± 22

[Protein]_50_ was calculated from the titration curves of EMSA by determining the concentration of the protein needed to achieve the midpoint value for input DNA binding. Errors are standard deviations determined by three independent titration experiments.

### Oligomeric state of SaSsbA in solution

**Fig 5:**
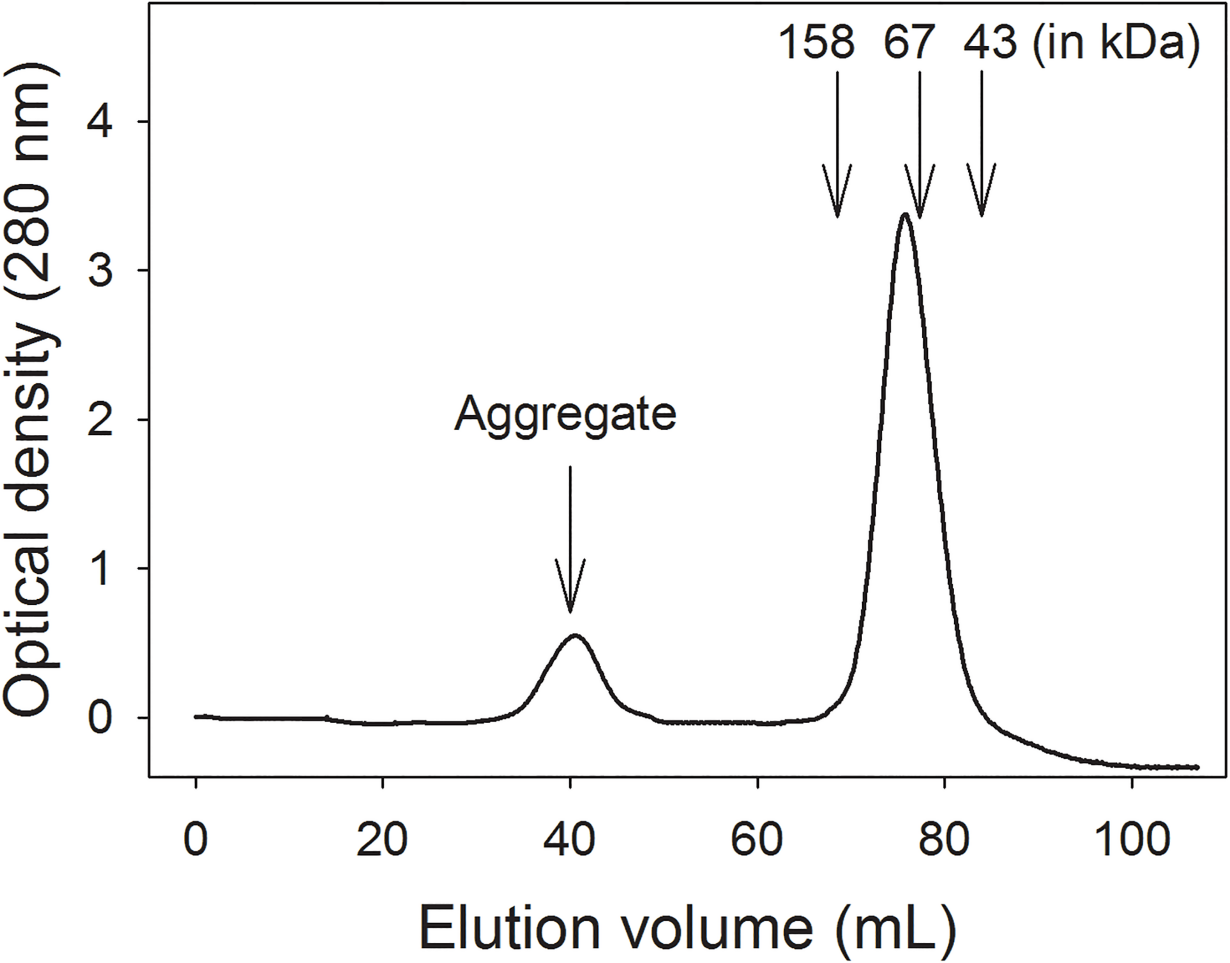
Oligomeric state of purified SaSsbA in solution. Purified protein in Buffer B was applied to a Superdex 200 prep grade column equilibrated with the same buffer. The column was calibrated with proteins of known molecular masses: thyroglobulin (670 kDa), γ-globulin (158 kDa), albumin (67 kDa), ovalbumin (43 kDa), chymotrypsinogen A (25 kDa) and ribonuclease A (13.7 kDa). The corresponding peak shows the eluted SaSsbA.

The oligomeric state of purified SaSsbA was analyzed by gel filtration chromatography (Fig 5), and the native molecular mass of SaSsbA was estimated to be 77 kDa. The native molecular mass for SaSsbA is approximately 4 times the molecular mass of a SaSsbA monomer (19 kDa). Thus, we concluded that SaSsbA in solution is a stable tetramer like EcSSB.

### SaSsbA cannot stimulate the ATPase activity of SaPriA

**Fig 6:**
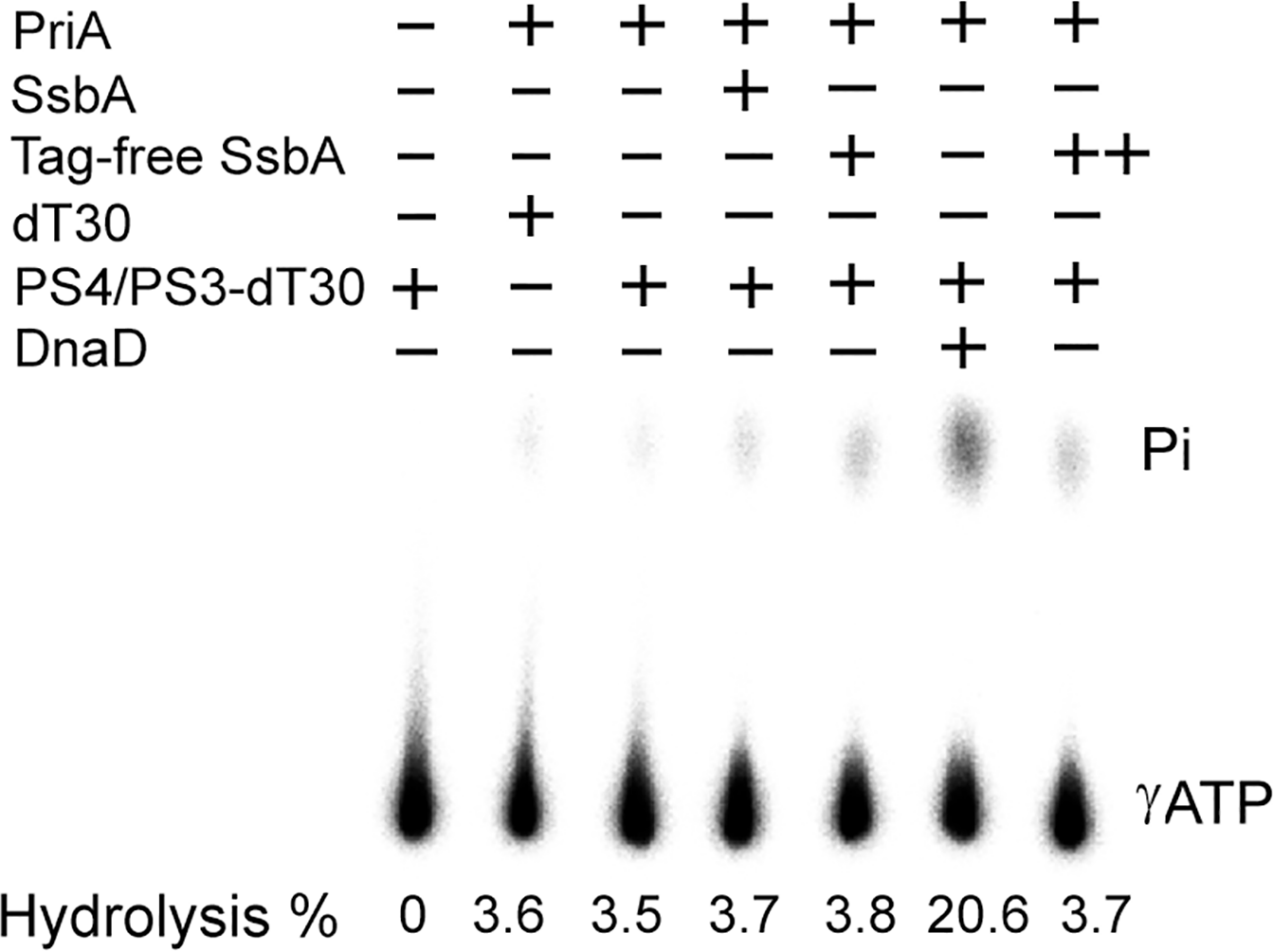
The ATPase activity of SaPriA did not change when acting with SaSsbA. SaPriA ATPase assay was performed with 0.4 mM [γ-32P] ATP, 0.12 μM of SaPriA, and 0.1 μM PS4/PS3-dT30 (or dT30) DNA substrate for 1 h. To study the effect, SaSsbA (10 μM), tag-free SaSsbA (10 μM), or SaDnaD (4 μM) was added into the assay solution. Higher concentration of tag-free SaSsbA (20 μM; denotes ++) was also used in this study. Aliquots (5 μL) were taken and spotted onto a polyethyleneimine cellulose thin-layer chromatography plate, which was subsequently developed in 0.5 M formic acid and 0.25 M LiCl for 30 m. Reaction products were visualized by autoradiography and quantified with a Phosphorimager.

PriA is known as a poor helicase when acting alone in vitro [72]. Gram-negative EcPriA activity can be significantly stimulated by PriB and SSB [46, 47]. Recently, we also found that SaDnaD can significantly enhance the activity of SaPriA [58]. Whether SaSsbA can stimulate SaPriA activity is unknown. SaPriA could hydrolyze ATP alone; however, no effect was found on the SaPriA activity when acting with SaSsbA (Fig 6). To exclude the possible effect of a His tag, tag-free SaSsbA was also used for this experiment, and a similar result was found. When tag-free SaSsbA was present at higher concentration (20 μM), a similar result was still found (Fig 6). Thus, in contrast to the case in EcPriA, SaSsbA does not affect the activity of SaPriA.

### Crystal structure of SaSsbA

**Fig 7:**
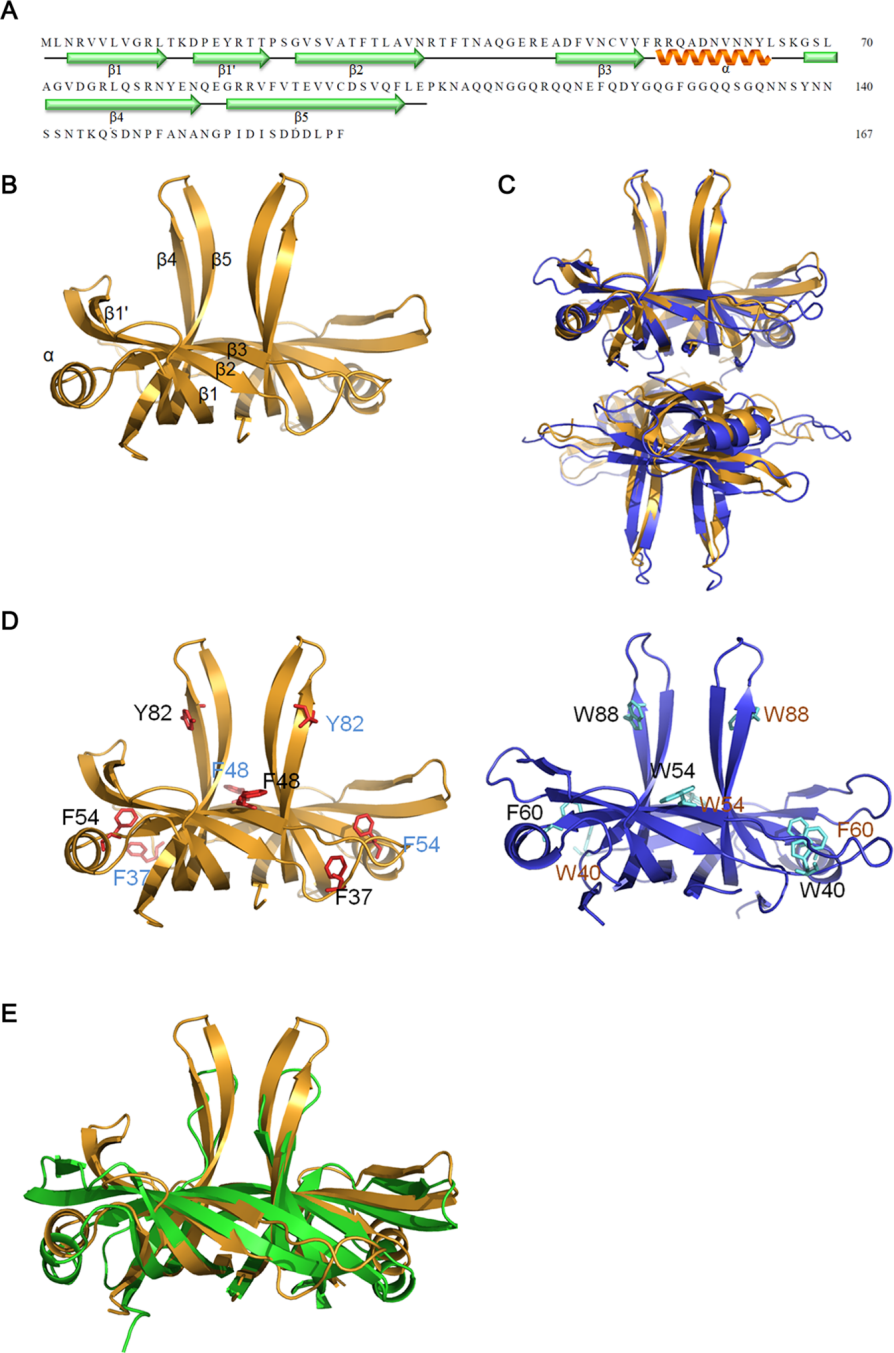
Crystal structure of SaSsbA. (A) The secondary structural element of SaSsbA. The secondary structural element of SaSsbA is shown above its sequence. (B) Crystal structure of SaSsbA. The cell unit contains two monomers of SaSsbA, and the core of the OB-fold possesses a β-barrel capped by an α-helix. In both subunits, the majority of the electron density exhibited good quality only for the N-terminal region of SaSsbA (aa 1–104); the aa 105–167 was unobserved. (C) Superposition of SaSsbA and EcSSB. The N-terminal domains of SaSsbA and EcSSB (PDB entry 1EYG; dark blue) are similar, but the structural differences are still found, the loops L_12_ and L_23_. Because the oligomerization state of SaSsbA in solution is tetrameric, SaSsbA may function as a tetramer like EcSSB. (D) ssDNA-binding mode of SaSsbA. In the EcSSB–ssDNA complex (PDB entry 1EYG), four essential aromatic residues, including Trp40, Trp54, Phe60, and Trp88, participate in ssDNA binding via stacking interactions. The structurally corresponding residues in SaSsbA are Phe37, Phe48, Phe54, and Tyr82. The ssDNA-binding mode of SaSsbA may be similar to that of EcSSB. (E) Superposition of SaSsbA and KpPriB. The N-terminal domain of SaSsbA and KpPriB (PDB entry 4APV; green) are similar, in which the only significant difference is in the lengths of the β4 and β5 sheets.

To obtain an in-depth understanding of the structure–function relationship of SaSsbA and of the explanation why it cannot produce the same effect on SaPriA as EcSSB did on EcPriA, we crystallized SaSsbA and determined its structure at a resolution of 1.82 Å (Table 3). The secondary structural element of SaSsbA is shown in Fig 7A. The cell unit contains two monomers of SaSsbA (Fig 7B), but its oligomerization state in solution is tetrameric (Figs 5 and 7C). The SaSsbA monomer has an OB-fold domain similar to EcSSB, and the core of the OB-fold possesses a β-barrel capped by an α-helix. In both subunits, the majority of the electron density, only for the N-terminal region of SaSsbA, exhibited good quality (aa 1–104); the aa 105–167 was unobserved. In the EcSSB–ssDNA complex, four essential aromatic residues, Trp40, Trp54, Phe60, and Trp88, participate in ssDNA binding via stacking interactions. The corresponding residues in SaSsbA are Phe37, Phe48, Phe54, and Tyr82, may play a similar role in ssDNA binding as EcSSB (Fig 7D). Although the N-terminal domains of SaSsbA and EcSSB are similar, their loops L_12_ and L_23_ are structurally different, one is short, and the other is extended (Fig 7C). Structurally, SaSsbA also resembled PriB (Fig 7E), in which the only significant difference is in the lengths of the β4 and β5 sheets.

**Table 3:** Data collection and processing statistics.

Data collection			
	Crystal	SaSsbA	
	Wavelength (*Å*)	0.975	
	Resolution (*Å*)	30–1.76	
	Space group	P4_1_2_1_2	
	Cell dimension (*Å*)	a = 88.792	α = 90
		b = 88.792	β = 90
		c = 57.686	γ = 90
Completeness (%)		99.8 (99.9)*	
<I/σI>		34.6 (3.2)	
*R*_sym_ or *R*_merge_ (%)		0.052 (0.559)	
Redundancy		8.6 (8.4)	
Refinement			
	Resolution (*Å*)	28.078–1.82	
	No. reflections	23454	
	*R*_work_/*R*_free_	0.1932/0.2233	
	No. atoms		
	Protein	209	
	Water	110	
R.m.s deviation			
	Bond lengths (*Å*)	0.0179	
	Bond angles (°)	1.8595	
Ramachandran Plot			
	In preferred regions	192 (93.66%)	
	In allowed regions	8 (3.9%)	
	Outliers	5 (2.44%)	
	PDB entry	5XGT	

*Values in parentheses are for the highest resolution shell.

Rsym = Σ|I − ‘I’ |/ΣI, where I is the observed intensity, ‘I’ is the statistically weighted average intensity of multiple observations of symmetry-related reflections.

### Conserved SSB-Ct binding site of PriA is not present in SaPriA

**Fig 8:**
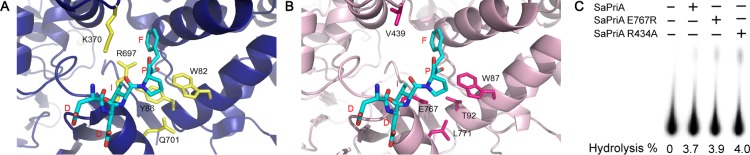
Conserved SSB-Ct binding site of PriA is not present in SaPriA. (A) SSB-Ct (DDIPF) binding site in KpPriA revealed by the complexed crystal structure (PDB ID: 4NL8). The KpPriA SSB-Ct binding site includes Trp82, Tyr86, Lys370, Arg697, and Gln701. (B) The putative SSB-Ct binding sites in SaPriA structurally corresponding with those in KpPriA are Trp89, Thr92, V439, Glu767, and Leu771. Only Trp89 in SaPriA (Trp82 in KpPriA) is conserved. Arg697, the most important residue in KpPriA in altering the SSB_35_/SSB_65_ distribution, is Glu767 in SaPriA. (C) Mutational analysis of SaPriA. The ATPase assay for the mutant SaPriA E767R and SaPriA R434A proteins was performed with 0.4 mM [γ-32P] ATP, 0.12 μM of the protein, SaSsbA (10 μM), and 0.1 μM PS4/PS3-dT30 DNA substrate for 1 h. Aliquots (5 μL) were taken and spotted onto a polyethyleneimine cellulose thin-layer chromatography plate, which was subsequently developed in 0.5 M formic acid and 0.25 M LiCl for 30 m. Reaction products were visualized by autoradiography and quantified with a Phosphorimager.

PriA interacts with the SSB-Ct at replication forks [47, 73, 74]. In addition, this interaction is a driving force to stimulate the PriA activity [47]. The crystal structure of KpPriA in complex with an SSB-Ct peptide, which was determined at 4.1 Å resolution, reveals a specific and conserved binding pocket in PriA [44]. The KpPriA SSB-Ct binding site includes Trp82, Tyr86, Lys370, Arg697, and Gln701, in which Arg697 is located near the α-carboxyl group of the C-terminal-most residue of the SSB-Ct (Fig 8A) and plays a critical role in altering the SSB_35_/SSB_65_ distribution [44]. Interestingly, however, we found that such an important residue in EcPriA, namely, Arg697, is not conserved (Fig 2). The corresponding residue in SaPriA is Glu767 instead (Fig 8B and S1 Fig), which could possibly be the reason for the inability of SaSsbA to stimulate SaPriA. To have an Arg residue in the SSB-Ct binding site of SaPriA as KpPriA, we then constructed and analyzed the mutant SaPriA E767R, but still produced a similar result as the wild-type SaPriA did (Fig 8C). Furthermore, we found another Arg (Arg434) near the SSB-Ct pocket in SaPriA, which may play a similar role as the Arg697 in EcPriA; however, no difference was observed in the ATPase activity for the mutant SaPriA R434A (Fig 8C). Thus, whether the SSB-Ct binding site in SaPriA contains Arg or not is not crucial for SaPriA activation by SaSsbA.

### SaPriA binds to SaSsbA, but not to the SSB-Ct peptide

**Fig 9:**
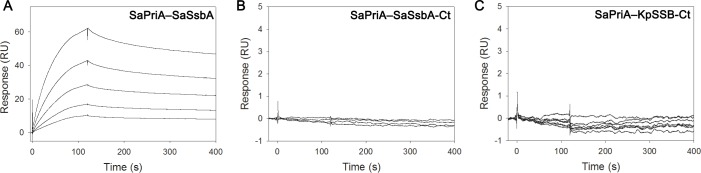
SPR analysis. (A) The SaPriA–SaSsbA interaction. SaPriA was immobilized on Series S sensor chips CM5, and the binding experiments were carried out using a Biacore T200. SaSsbA (1000, 500, 250, 125, and 63 nM) was injected in duplicate over the immobilized protein for 120 s at a flow rate of 30 μL/min. The estimated *K*_d_ value was derived by fitting the association and dissociation signals with a 1:1 (Langmuir) model using the Biacore T200 Evaluation Software. (B) The SaPriA–SaSsbA-Ct interaction analyzed by SPR. (C) The SaPriA–KpSSB-Ct interaction analyzed by SPR.

EcPriA is known to bind SSB-Ct [47], and complexed crystal structure of KpPriA further showed this binding pocket [44]. In this study, we found that the binding site of KpPriA to SSB-Ct is not found in SaPriA (Fig 8), and SaSsbA failed to stimulate SaPriA (Fig 6). To confirm whether SaPriA interacts with SaSsbA, we used SPR to provide experimental evidence for estimating the binding affinity between these proteins and thus quantitatively characterize this interaction further (Fig 9). SaPriA was immobilized on a sensor chip (as a ligand), and the SaSsbA solution (as an analyte) was passed over the sensor chip in a microfluidic chamber. Fig 9A shows the SPR results at various SaSsbA concentrations. The *K*_d_ value of SaPriA bound by SaSsbA, calculated from the equilibrium binding isotherms using a simple binding model (a 1:1 Langmuir binding model), was 4.6 ± 0.5 × 10^−8^ M. Thus, even no identical binding site as in KpPriA, SaPriA can bind to SaSsbA.

To determine whether SaPriA can bind to SaSsbA-Ct (NANGPIDISDDDLPF) and KpSSB-Ct (PSNEPPMDFDDDIPF), chemically synthesized SaSsbA-Ct and KpSSB-Ct were used for SaPriA-binding experiments. SaSsbA-Ct was injected at increasing concentrations, but the binding response for SaPriA did not change, indicating no interaction (Fig 9B). No interaction of KpSSB-Ct with SaPriA was also found (Fig 9C). Therefore, unlike EcPriA, which binds to EcSSB-Ct directly, SaPriA cannot bind to SaSsbA-Ct and KpSSB-Ct.

### KpSSB, but not KpSSB-Ct, can significantly stimulate the ATPase activity of SaPriA

**Fig 10:**
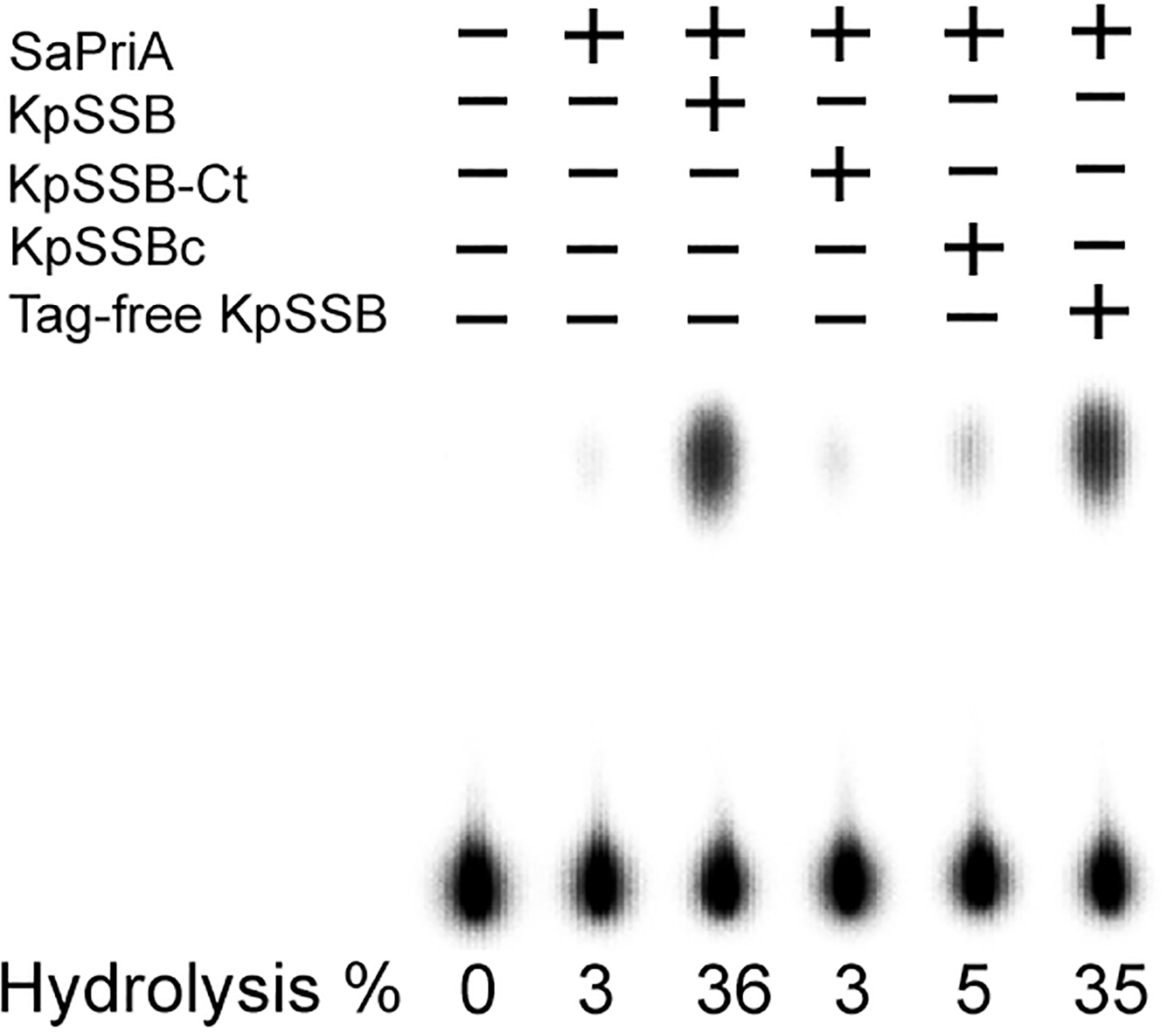
KpSSB, but not KpSSB-Ct, can significantly stimulate the ATPase activity of SaPriA. SaPriA ATPase assay was performed with 0.4 mM [γ-32P] ATP, 0.12 μM of SaPriA, and 0.1 μM PS4/PS3-dT30 DNA substrate for 1 h. To study the effect, the protein (10 μM) was individually added into the assay solution. Aliquots (5 μL) were taken and spotted onto a polyethyleneimine cellulose thin-layer chromatography plate, which was subsequently developed in 0.5 M formic acid and 0.25 M LiCl for 30 m. Reaction products were visualized by autoradiography and quantified with a Phosphorimager.

The typical SSB-Ct binding site of PriA in SaPriA is not conserved (Fig 8). To investigate whether all SSB cannot stimulate SaPriA like SaSsbA, the ATPase activity of SaPriA was assayed in the presence of KpSSB [60]. Tag-free KpSSB [61] was also used for this cross-species analysis. As shown in Fig 10, SaPriA ATPase activity was significantly stimulated (twelvefold) when acting with either KpSSB or tag-free KpSSB. Chemically synthesized KpSSB-Ct (the last 15 amino acids of the KpSSB C terminus) was also used for this analysis and was found to fail to stimulate SaPriA. To determine the possible role of the flexible region, KpSSBc, the C-terminal domain of KpSSB that comprises the flexible region and SSB-Ct (aa 116–174), was also analyzed. When acting with KpSSBc, a slight stimulation of SaPriA ATPase (less than twofold) was observed. Thus, we conclude that KpSSB and KpSSBc, except KpSSB-Ct, exhibited a cross-species stimulation effect for SaPriA.

### S161 in SaSsbA determines the stimulation property of SaPriA

**Fig 11:**
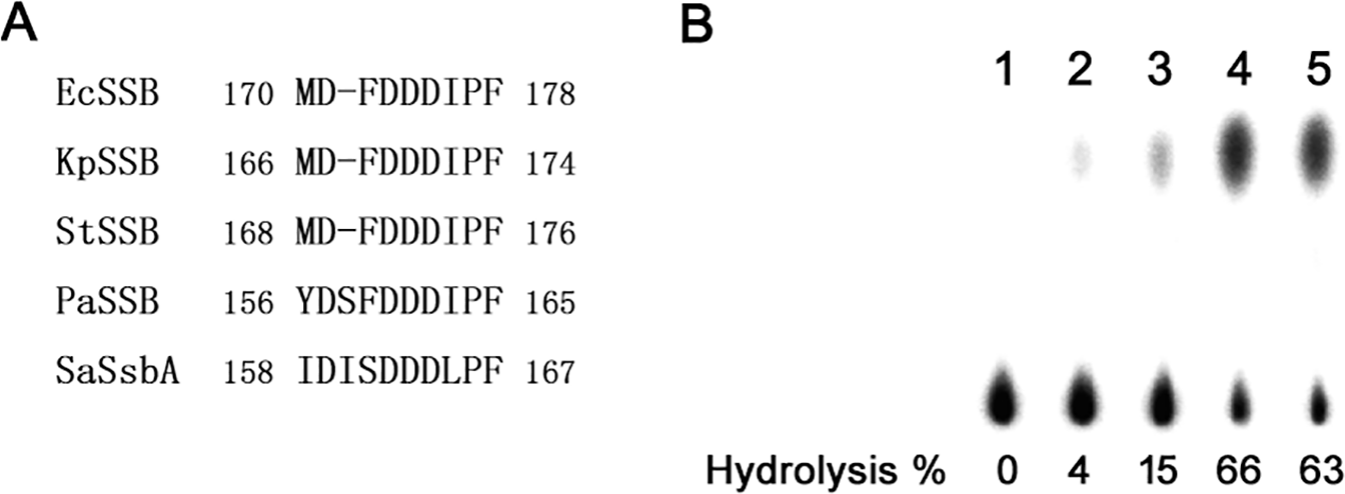
S161 in SaSsbA is a switch for SaPriA stimulation. (A) Multiple amino acid sequence alignment of SSB-Ct from Ec, Kp, St, Pa, and Sa. FDDDIPF in the C-terminal domain of SSB is usually conserved, but is not for SaSsbA. (B) SaPriA ATPase assay was performed with 0.4 mM [γ-32P] ATP, 0.12 μM of SaPriA, and 0.1 μM PS4/PS3-dT30 DNA substrate for 1 h. To study the effect, the mutant protein was individually added into the assay solution. Aliquots (5 μL) were taken and spotted onto a polyethyleneimine cellulose thin-layer chromatography plate, which was subsequently developed in 0.5 M formic acid and 0.25 M LiCl for 30 m. Reaction products were visualized by autoradiography and quantified with a Phosphorimager. Reaction was carried out without SaPriA (lane 1) or with SaPriA alone (lane 2) as controls. SaPriA acted with 5 μM SaSsbA plus 5 μM SaSsbA S161F mutant (lane 3), 10 μM SaSsbA S161F (lane 4), and 10 μM SaSsbA S161F/delI160 double mutant (lane 5) as shown, respectively.

The N-terminal domain of SSB proteins is basically conserved in sequence and structure [1, 2], such as those in SaSsbA and EcSSB (Fig 7). As the stimulation of SaPriA was found by acting with KpSSB (Fig 10), but not SaSsbA (Fig 6), we further investigated whether the different SSB-Ct is related to the stimulation switch. We compared the sequence of the C-terminal acidic tails of SSBs and found that the conserved MDFDDDIPF motif in the Gram-negative bacterial SSB, such as Ec, Kp, and St, in which whose motif is DISDDDLPF in SaSsbA, i.e., the amino acid residue F172 in EcSSB and F168 in KpSSB, whose corresponding residue in SaSsbA is S161, not F. To investigate the role of the conserved F residue in SSB proteins (but the residue is S in SaSsbA) in stimulation of PriA activity, SaSsbA S161F mutant was accordingly created with F substitution, and ATPase activity of SaPriA was then assessed (Fig 11). Unlike the wild-type protein, SaPriA ATPase activity was dramatically stimulated when acting with SaSsbA S161F mutant (elevenfold), suggesting that the residue F is important in PriA stimulation. According to sequences, we also constructed the double mutant SaSsbA (S161F/delI160) for analysis with SaPriA; this double mutant, in addition to S161F, whose I160 residue was also deleted. When acting with SaSsbA S161F/delI160 mutant, SaPriA ATPase activity was still stimulated as that with SaSsbA S161F mutant, suggesting a no/minor role of the I160 in PriA stimulation. Overall, based on results from the sequence comparison for the C terminal region in SSB proteins (Fig 11B and S2 Fig) and the ATPase assay, we conclude that S161 in SaSsbA is a switch for SaPriA stimulation. However, it is unclear why the residue F, which is conserved in SSB proteins from the Gram-negative bacteria, is changed to S in SaSsbA.

## Discussion

It is believed that all cells present now evolved from a common ancestor, implying that the basic principles learned from experiments performed with one type of cell should be generally applicable to other cells. Given the comparative simplicity, *E*. *coli* has long been the favored organism for studying many fundamental aspects of biochemistry and molecular biology. However, because of many obvious differences in the mechanisms of action of DNA replication restart primosome found between *E*. *coli* and Gram-positive bacteria [55], the process by which PriA can cooperate with different loading factors to reactivate same stalled forks still need to be elucidated [43, 75]. Gene map analysis shows that unlike *E*. *coli ssb* gene located adjacent to *uvrA* gene, *S*. *aureus ssb* gene (SaSsbA) is flanked by the *rpsF* and *rpsR* genes, coding for the ribosomal proteins S6 and S18, respectively (Fig 1). In this study, we characterized *S*. *aureus* main SSB and found that it cannot stimulate PriA (Fig 6), unlike *E*. *coli* [47]. In addition, we also found that the 15 C-terminal amino acids of *E*. *coli* SSB, known to bind EcPriA, did not bind to SaPriA demonstrated using SPR (Fig 9); that is, in the results of this present study are not in agreement with those for *E*. *coli*. Whether these significant disparities are due to inherent differences among the species, the use of different assay methods, or the effect of different investigators, remains unknown. However, we also noted that these seemingly contradictory data may reconcile. Our structure-sequence comparison (Figs 7 and 8) and mutational analysis (Fig 11) further indicates that the PriA SSB-Ct binding site (Trp82, Tyr86, Lys370, Arg697, and Gln701), revealed by a 4.1 Å resolution crystal structure [44], is not applicable to SaPriA (Fig 8). Based on these results, we conclude that SaSsbA may bind SaPriA in a different manner compared with that of EcSSB-EcPriA. Whether SaSsbA cannot stimulate SaPriA activity because of this different binding mechanism still remains to be exploited.

The SSB-Ct involved in protein-binding during DNA metabolism is known as the SSB interactome [2]. Studies both in vivo and in vitro using the C-terminal deletion mutants of SSB have found that the last eight residues are important for binding to the target protein, such as RecG and PriA [4, 47, 76–78]. Recently, however, detailed analyses indicate that PXXP motifs in the intrinsically disordered linker (IDL) of SSB are directly responsible for mediating the protein–protein interactions; removal of the last eight residues of SSB with negative effect on partner protein binding may not be a correct indication of protein–protein interaction site [4, 79, 80]. In this study, both SaSsbA-Ct and KpSSB-Ct peptides did not bind to SaSsbA (Fig 9); thus, we also try to find whether the PXXP motifs are present in SaSsbA. However, unlike EcSSB [4], KpSSB [60], StSSB [81] and PaSSB [82] contain many Pro residues in their C-terminal domains [61], SaSsbA contains very few Pro residues (Fig 3). In addition, their positions in SaSsbA are not able to match the PXXP motif [4]. Whether the Gram-positive and Gram-negative SSB use a general mechanism to bind their partner proteins during DNA metabolism is still unclear.

Almost non-hexameric helicases have poor dsDNA unwinding activities when acting alone in vitro [72]. Recently, the first case for the Gram-positive bacterial PriA activity stimulation has been reported: SaDnaD can obviously enhance the ATPase activity of SaPriA [58]. Like EcSSB, SaSsbA, and EcPriB, SaDnaD can bind ssDNA and PriA [58, 83]. Given that SaSsbA and SaDnaD are both ssDNA- and SaPriA-binding proteins, and both function in early steps of the Gram-positive primosome assembly [75, 84], more studies are still needed to determine whether or not SaSsbA is a competitor or an enhancer for SaDnaD binding to PriA, and PriA bound forked DNA.

## Supporting information

S1 Fig Multiple amino acid sequence alignment of PriA proteins. Sequence alignment of SaPriA, KpPriA, and EcPriA was generated by CLUSTALW2. The KpPriA SSB-Ct binding sites (Trp82, Tyr86, Lys370, Arg697, and Gln701) are colored in red.
(TIF)

S2 Fig Multiple amino acid sequence alignment of SSB-Ct from some Gram-positive bacteria. Including *Bacillus subtilis*, IDISDDDLPF in the C-terminal domain of SSB from the Gram-positive bacteria is usually conserved.
(TIF)
