# Supplementary figures and images for: Staphylococcus aureus single-stranded DNA-binding protein SsbA can bind but cannot stimulate PriA helicase
Source: PLoS One. 2017 Jul 27;12(7):e0182060. doi: 10.1371/journal.pone.0182060 (PMC5531588; doi:10.1371/journal.pone.0182060)

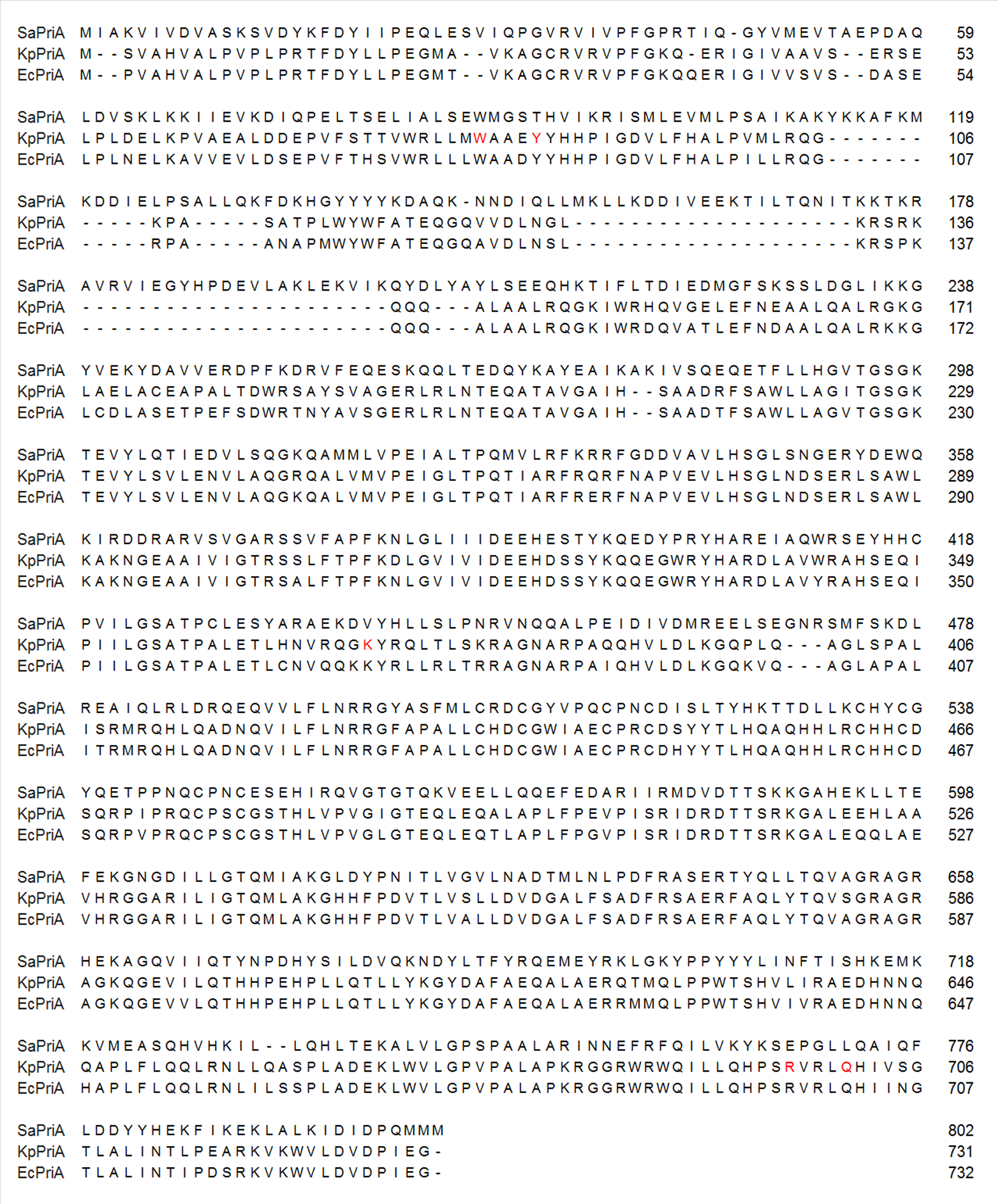

Supplement: S1 Fig — Sequence alignment of SaPriA, KpPriA, and EcPriA was generated by CLUSTALW2. The KpPriA SSB-Ct binding sites (Trp82, Tyr86, Lys370, Arg697, and Gln701) are colored in red. (TIF) [file pone.0182060.s001.tif]

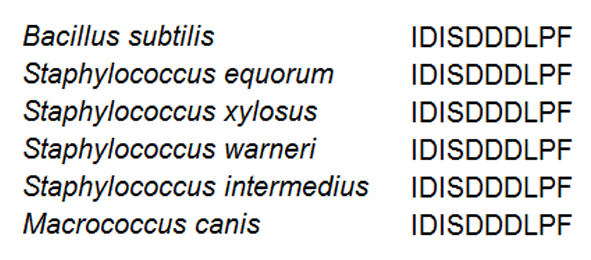

Supplement: S2 Fig — Including Bacillus subtilis, IDISDDDLPF in the C-terminal domain of SSB from the Gram-positive bacteria is usually conserved. (TIF) [file pone.0182060.s002.tif]
